# Madagascar Leaf-Tail Geckos (*Uroplatus* spp.) Share Independently Evolved Differentiated ZZ/ZW Sex Chromosomes

**DOI:** 10.3390/cells12020260

**Published:** 2023-01-09

**Authors:** Eleonora Pensabene, Alona Yurchenko, Lukáš Kratochvíl, Michail Rovatsos

**Affiliations:** Department of Ecology, Faculty of Science, Charles University, 128 00 Prague, Czech Republic

**Keywords:** DNA-seq, genomics, cytogenetics, evolution, reptiles, sex chromosomes, sex determination, qPCR

## Abstract

Geckos are an excellent group to study the evolution of sex determination, as they possess a remarkable variability ranging from a complete absence of sex chromosomes to highly differentiated sex chromosomes. We explored sex determination in the Madagascar leaf-tail geckos of the genus *Uroplatus*. The cytogenetic analyses revealed highly heterochromatic W chromosomes in all three examined species (*Uroplatus henkeli*, *U. alluaudi*, *U. sikorae*). The comparative gene coverage analysis between sexes in *U. henkeli* uncovered an extensive Z-specific region, with a gene content shared with the chicken chromosomes 8, 20, 26 and 28. The genomic region homologous to chicken chromosome 28 has been independently co-opted for the role of sex chromosomes in several vertebrate lineages, including monitors, beaded lizards and monotremes, perhaps because it contains the *amh* gene, whose homologs were repeatedly recruited as a sex-determining locus. We demonstrate that all tested species of leaf-tail geckos share homologous sex chromosomes despite the differences in shape and size of their W chromosomes, which are not homologous to the sex chromosomes of other closely related genera. The rather old (at least 40 million years), highly differentiated sex chromosomes of *Uroplatus* geckos can serve as a great system to study the convergence of sex chromosomes evolved from the same genomic region.

## 1. Introduction

Vertebrates possess variability in sex determination ranging from environmental sex determination (ESD), where there are no consistent differences in genotype between sexes, to genotypic sex determination (GSD) with highly differentiated sex chromosomes [1,2,3,4]. However, this variability is distributed highly unequally among clades. Some lineages, such as viviparous mammals [5], birds [6], iguanas [7], caenophidian snakes [8], monitor lizards [9], skinks [10], lacertid lizards [11], certain lineages of teleosts [12,13] or sturgeons [14] have stable sex chromosomes for dozens of millions of years. On the other hand, frequent transitions, usually turnovers of sex chromosomes, are common in other lineages, for example, ranid frogs [15], salmonids [16,17], sticklebacks [18] and medakas [19], with the most extreme rate of turnovers being documented in cichlids [20]. The reasons for this variability are not yet totally elucidated [3].

Among amniotes, geckos are probably the most variable clade in sex determination [21,22,23]. Geckos are an ancient, highly diversified group (>2000 described species) [24]. They are likely sister to all other squamate reptiles with the exception of dibamids [25]. The variability in sex determination among geckos can reflect their ancient origin and independent emergences of GSD from putative ancestral ESD [23,26,27], but also turnovers of sex chromosomes as documented, for example, within the genera *Coleonyx*, *Sphaerodactylus* and *Cyrtodactylus* [28,29,30,31]. However, certain gecko lineages, such as the legless family Pygopodidae and the family Carphodactylidae, have old sex chromosomes, stable for 32–72 MY and 15–45 MY, respectively [21,32]. The contribution of the mechanisms generating the variability in the whole gekkotan clade and stability in some subclades is difficult to disentangle, as sex determination has remained poorly studied or unstudied in many lineages.

Here, we focused on the Madagascar leaf-tail geckos of the genus *Uroplatus* (family Gekkonidae). This notable genus includes more than 20 currently recognized middle to large bodied species of geckos with well-developed camouflage to resemble tree barks [33]. Madagascar leaf-tailed geckos are nested inside the species-rich gekkotan family Gekkonidae (more than 1500 species) [24], where XX/XY and ZZ/ZW sex chromosomes evolved multiple times among (and sometimes within) the genera *Gekko* [34,35,36,37,38], *Paroedura* [23,39,40], *Cyrtodactylus* [29,41], *Heteronotia*, *Hemidactylus*, *Gehyra* and *Dixonius* [23,42,43], but ESD was identified in the genus *Phelsuma* (reviewed in [21,23]). Sex-determination systems are under debate or totally unstudied in many other gekkonid species, including the Madagascar leaf-tail geckos. Very recently, Mezzasalma et al. [44] performed the first cytogenetic analysis in eight species of the genus *Uroplatus*. They found a highly heteromorphic pair with a distinctly heterochromatic chromosome in a female of *U. alluaudi,* suggesting it is a ZW pair of sex chromosomes. Unfortunately, a male was not available in this study to check if this heteromorphism is sex-specific, and no heteromorphism was noticed in *U. ebenaui*, the only other species where a female individual was examined. Mezzasalma et al. [44] suggested a relatively recent origin of the differentiated ZZ/ZW sex chromosomes within the genus, as they found the heteromorphic chromosome pair only in *U. alluaudi*. The recent origin was also expected from a large size of the putative W, as the authors assumed that an expansion of unpaired sex chromosomes is an indication of its relatively recent diversification by heterochromatin addition and amplification, which should be followed by size shrinkage at later stages. Based on chromosome morphology, Mezzasalma et al. [44] also suggested that these putative sex chromosomes evolved independently of the ZZ/ZW sex chromosomes of the relatively closely related Madagascar geckos of the genus *Paroedura* [39,40].

Here, we tested whether differentiated ZZ/ZW sex chromosomes are indeed present in the members of the genus *Uroplatus*. Next, we identified the partial gene content of the sex chromosomes and tested more rigorously by a comparison of gene content, whether they are homologous to the sex chromosomes of other geckos.

## 2. Materials and Methods

### 2.1. Studied Material

Blood samples were collected from 13 species of geckos, including five species of Madagascar leaf-tail geckos (list of species in Table 1). Geckos are prone to shed tail by autotomy; therefore, we collected a blood sample from the brachial vein of the front legs using an insulin-type syringe with 50 μL of heparin solution (5000 IU/mL; Zentiva, Prague, Czech Republic) instead of the caudal vein. In addition, the tip of the tail was collected from frozen specimens of *Uroplatus phantasticus*. All experimental procedures were carried out under the supervision and with the approval of the Ethics Committee of the Faculty of Science, Charles University, followed by the Committee for Animal Welfare of the Ministry of Agriculture of the Czech Republic (permissions No. 35484/2015-14 and 8604/2019-7).

### 2.2. Preparation of Chromosome Suspensions

Chromosome suspensions were prepared from whole-blood cell cultures in three species of the Madagascar leaf-tail geckos (*U. alluaudi, U. henkeli, U. sikorae*), following the protocol described in [45]. Briefly, the culture medium consisted of 90 mL of DMEM medium (Sigma-Aldrich, St. Louis, MO, USA) enriched with 10 mL of fetal bovine serum (GIBCO), 3 mL of phytohemagglutinin M (GIBCO), 1 mL of penicillin/streptomycin solution (10,000 units/mL; GIBCO, Waltham, MA, USA), 1 mL of L-glutamine solution (200 mM; Sigma-Aldrich) and 1 mL of lipopolysaccharide solution (10 mg/mL; Sigma-Aldrich). Subsequently, 100–300 μL of blood was added to 5 mL of cultivation medium and incubated at 30 °C for one week. After the incubation period, we used 35 μL of colcemid solution (10 μg/mL; Roche, Basel, Switzerland) to block cell division and then incubated the cultures for 3 h and 30 min at 30 °C. Subsequently, the cells were treated with a pre-warmed hypotonic solution (0.075 M KCl) for 30 min at 37 °C, washed by centrifugation at 800–1200 rpm for 10 min, and fixed four times with cold 3:1 methanol/acetic acid solution for 20 min each, with intermediate centrifugation at 1200 rpm for 10 min. Chromosome suspensions were spread onto slides and incubated at 60 °C for 1 h, prior to all cytogenetic experiments. The remaining chromosome suspensions were stored at −20 °C.

### 2.3. Chromosome Staining and Karyotype Reconstruction

The slides were stained with 5% Giemsa solution. Selected metaphases were captured using a Zeiss Axio Imager Z2 (Zeiss, Oberkochen, Germany), equipped with a Metafer-MSearch automatic scanning platform (MetaSystems) and CoolCube 1 b/w digital camera (MetaSystems). Karyograms were prepared using the Ikaros karyotyping platform (MetaSystems). At least 10 metaphases per individual were studied. We performed C-banding stain to detect heterochromatin distribution according to the protocol of Sumner [46]. The slides were first treated with 0.2 N HCl for 30 min at room temperature, then with prewarmed 5% Ba(OH)_2_ for 10 min at 45 °C and finally in 2× SSC for 1 h at 60 °C. Subsequently, the slides were washed with distilled water, air-dried and stained with Fluoroshield mounting medium with DAPI stain (Sigma-Aldrich).

### 2.4. Fluorescence In Situ hybridization with Probe for Telomeric Sequences, 18S/28S rDNA Loci and GATA Microsatellite Motif

The telomeric probe (TTAGGG)_n_ was prepared by Polymerase Chain Reaction (PCR) without a DNA template using the primers (TTAGGG)_5_ and (CCCTAA)_5_ (based on [47]). The probe was precipitated overnight at −20 °C in sodium acetate (3M, Saint Paul, MN, USA), sonicated with salmon sperm DNA, and 2.5 volumes of cold 100% ethanol and subsequently diluted in 50% formamide in 2× SSC (pH 7). The probe for rDNA loci was prepared according to the protocol of Rovatsos et al. [48], using a plasmid (pDmr.a 51#1) with an 11.5-kb insert encoding the 18S and 28S ribosomal units of *Drosophila melanogaster* [49]. The probe was labeled with dUTP-biotin (Roche, Basel, Switzerland) by a Nick Translation Kit (Abbott Laboratories, Lake Bluff, IL, USA) using the manufacturer’s protocol. The probe for the (GATA)_8_ microsatellite motif was commercially synthesized and terminally labeled with biotin by Macrogen (Seul, South Korea).

The FISH experiments were conducted in two days, following the protocol described in [45]. Briefly, the slides were treated with RNAse A (100 μg/mL) for 1 h at 37 °C, 0.01% pepsin for 10 min at 37 °C, post-fixed in 1% formaldehyde solution for 10 min and dehydrated in 70–85–95% ethanol series, for 5 min each. Once dried, the slides were denatured in 70% formamide for 3 min at 75 °C and dehydrated once more in ethanol series. In the meanwhile, the probe was denatured for 6 min at 73 °C and kept on ice for at least 10 min. The probe was applied to the slides for overnight hybridization at 37 °C. During the second day, the slides were washed 3 times with 50% formamide/2× SSC solution for 5 min at 37 °C, two times with 2× SSC for 5 min and once with 4× SSC/0.05% Tween20 (Sigma-Aldrich) for 5 min. The slides were incubated in 4× SSC/5% blocking reagent (Roche) for 45 min at 37 °C and then in 4× SSC/5% blocking reagent containing avidin-FITC (Vector laboratories, Newark, CA, USA) for 30 min at 37 °C. The fluorescence signal was amplified twice by the avidin-FITC/biotinylated anti-avidin system (Vector Laboratories). After this treatment, the slides were dehydrated in ethanol series, air-dried and stained with Fluoroshield mounting medium with DAPI (Sigma-Aldrich). Photos were captured by an Olympus BX53 digital upright fluorescence microscope equipped with a 21-megapixel high-resolution digital DP74 color camera (Olympus, Shinjuku City, Japan).

### 2.5. Comparative Gene Coverage Analysis

Genomic DNA was extracted from blood samples from one male and one female specimen of Henkel’s leaf-tailed gecko *U. henkeli*, using the DNeasy Blood and Tissue Kit (Qiagen, Hilden, Germany), following the manufacturer’s protocol. The extracted DNAs were sequenced at Novogene (Cambridge, UK) on the Illumina HiSeq2500 platform, with 350 base pairs (bp) pair-end option. The raw Illumina reads were deposited in the NCBI database under the BioProject ID PRJNA917835. Adapters and low-quality bases were trimmed using Trimmomatic [50] and reads shorter than 50 bp were removed from further analyses. The trimmed Illumina reads were mapped to a reference data set of 170,981 exons, extracted from the *Gekko japonicus* genome project [51], using Geneious Prime v2022 (https://www.geneious.com, accessed on 2 May 2022). Subsequently, we estimated the average read coverage per gene in each specimen, and we calculated the ratio of female-to-male read coverage for each gene, normalized to the total number of assembled reads per specimen. Mapping parameters (Table S1) and additional methodological information were previously published in [10,21]. The Z-specific loci are expected to have half-read coverage in ZW females in comparison to ZZ males (i.e., a female-to-male ratio of 0.5), while autosomal, pseudoautosomal, and poorly differentiated loci should have equal read coverage in both sexes (i.e., a female-to-male ratio of 1.0).

We also identified the presence/absence of single-nucleotide polymorphisms (SNPs) per gene in each sex in order to further validate the Z-specific genes that were identified by the comparative gene coverage analysis. Z-specific, single-copy genes are hemizygous in ZW females. Therefore, such genes should lack SNPs in the map-to-reference assembly from the female specimen.

### 2.6. qPCR Validation of Z-Specific Genes and Test of Homology Across Geckos

ZW females should have half the number of Z-specific gene copies in their genome compared to ZZ males. This difference in gene copies between sexes can be estimated by qPCR; for a detailed methodology, check Refs. [7,52]. This qPCR-based approach facilitates the validation of the Z-specificity of genes, which were already identified by the gene coverage analysis in *U. henkeli*. Z-specific genes are expected to have a female-to-male ratio (*r*) in gene copy number of 0.5, while 1.0 is expected for autosomal or pseudoautosomal genes. With the same reasoning, we applied this qPCR-based approach to test if the genes Z-specific in *U. henkeli* are located to the differentiated sex chromosomes in other species of Madagascar leaf-tail geckos as well as in closely related genera of geckos in order to estimate the homology of sex chromosomes and the age of sex determination system in a phylogenetic context. The minimal age for sex chromosomes was determined as the estimated age of the last common ancestor of species sharing the same Z-specific genes from phylogenetic studies with molecular dating [53].

We designed primers for genes revealed to be Z-specific from the comparative gene coverage analysis in Primer-Blast software [54] using Primer3 [55]. In addition, we used previously designed primers for the autosomal genes *mecom, noct,* and *rag1* [40], which were used for the normalization of the qPCR quantification values (crossing point—cp) and for autosomal controls.

## 3. Results

### 3.1. Cytogenetic Analysis

**Figure 1:**
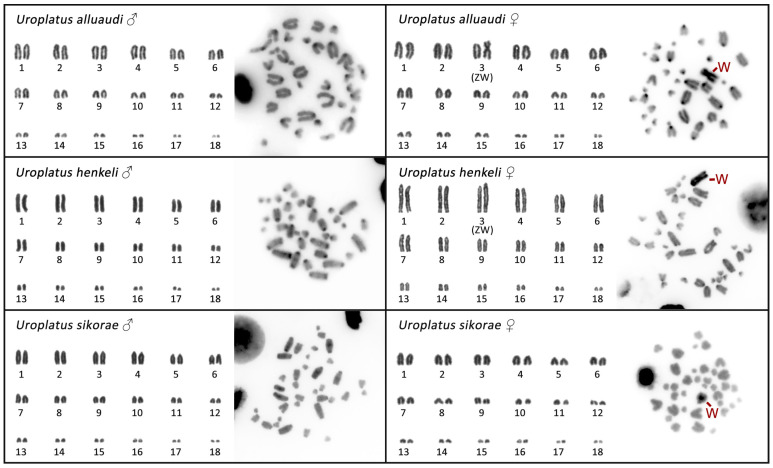
Karyograms and C-banded metaphases from both sexes of the Madagascar leaf-tail geckos (*U. alluaudi, U. henkeli* and *U. sikorae*). Sex chromosomes are indicated when we were able to identify them.

All three cytogenetically analyzed species of the Madagascar leaf-tail geckos (*U. alluaudi, U. henkeli, U. sikorae*) have a karyotype of 2n = 36 acrocentric chromosomes, gradually decreasing in size (Figure 1). The only exception is a submetacentric chromosome in the female *U. alluaudi*, which is not present in the karyotype from the male of the same species (Figure 1). 

C-banding revealed heterochromatin mainly in the centromeric regions of all chromosomes. An extensive accumulation of heterochromatin was detected in a single macrochromosome exclusively in the females of all three species (Figure 1). We conclude that this heterochromatic chromosome (large-sized metacentric in *U. alluaudi*, and large-sized acrocentric in *U. henkeli* and *U. sikorae*) should correspond to the W chromosome. The Z chromosome cannot be directly distinguished by morphology, as all the autosomes and the Z are acrocentric chromosomes gradually decreasing in size, but by karyogram reconstruction, we can assign the Z chromosome as the 3rd (or putatively the 4th) pair of the complement in *U. alluaudi* and *U. henkeli* (Figure 1).

**Figure 2:**
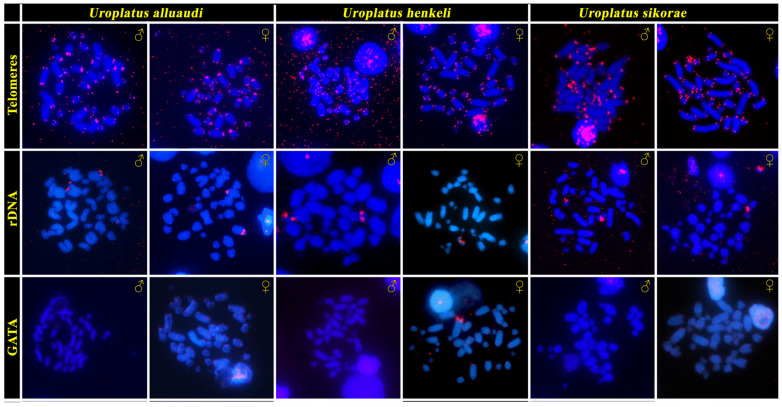
In situ hybridization with probes specific for telomeric repeats, rDNA loci and GATA microsatellite motifs in chromosome spreads from both sexes of the Madagascar leaf-tail geckos (*U. alluaudi, U. henkeli* and *U. sikorae*).

Telomeric motifs were observed in the expected terminal position of all chromosomes (Figure 2). A strong accumulation of rDNA loci was detected on a single pair of large-sized chromosomes in *U. alluaudi,* but in a pair of small-sized chromosomes in *U. henkeli* and *U. sikorae* (Figure 2). Neither telomeric motifs nor rDNA loci show a sex-specific pattern in all three examined species. FISH with a probe specific for the GATA microsatellites motif revealed a strong accumulation at the terminal position of a single macrochromosome in the female *U. henkeli*, which probably corresponds to the W chromosome (Figure 2).

### 3.2. Comparative Gene Coverage Analysis

**Figure 3:**
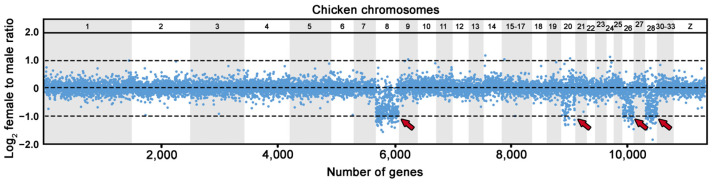
Log_2_-transformed female to male ratios of DNA-seq read coverage per gene in *U. henkeli.* Due to the lack of a chromosome-level genome assembly for *U. henkeli*, genes are illustrated based on the position of their orthologs in the chicken genome. The Z-specific genes are expected to show half female to male read coverage ratio (log_2_-transformed ratios of ∼−1.00) than autosomal and pseudoautosomal genes (log_2_-transformed ratios of ∼0.00). Genomic regions with Z-specific genes in *U. henkeli* (GGA8, GGA20, GGA26, GGA28) are indicated by arrows (all data are available in Table S2).

We analyzed 18.964 genes by comparative gene coverage analysis, revealing 432 genes with female-to-male gene read coverage between 0.35 and 0.65, and the absence of SNPs in at least 80% of the exons per gene (Figure 3; Table S1). Among these 432 putative Z-specific genes, 276 genes have known homologs in the chicken genome, linked mainly to the chicken chromosomes 8 (158 genes), 20 (19 genes), 26 (46 genes) and 28 (47 genes).

### 3.3. qPCR Validation of Z-Specific Markers and Test of Homology Across Geckos

**Figure 4:**
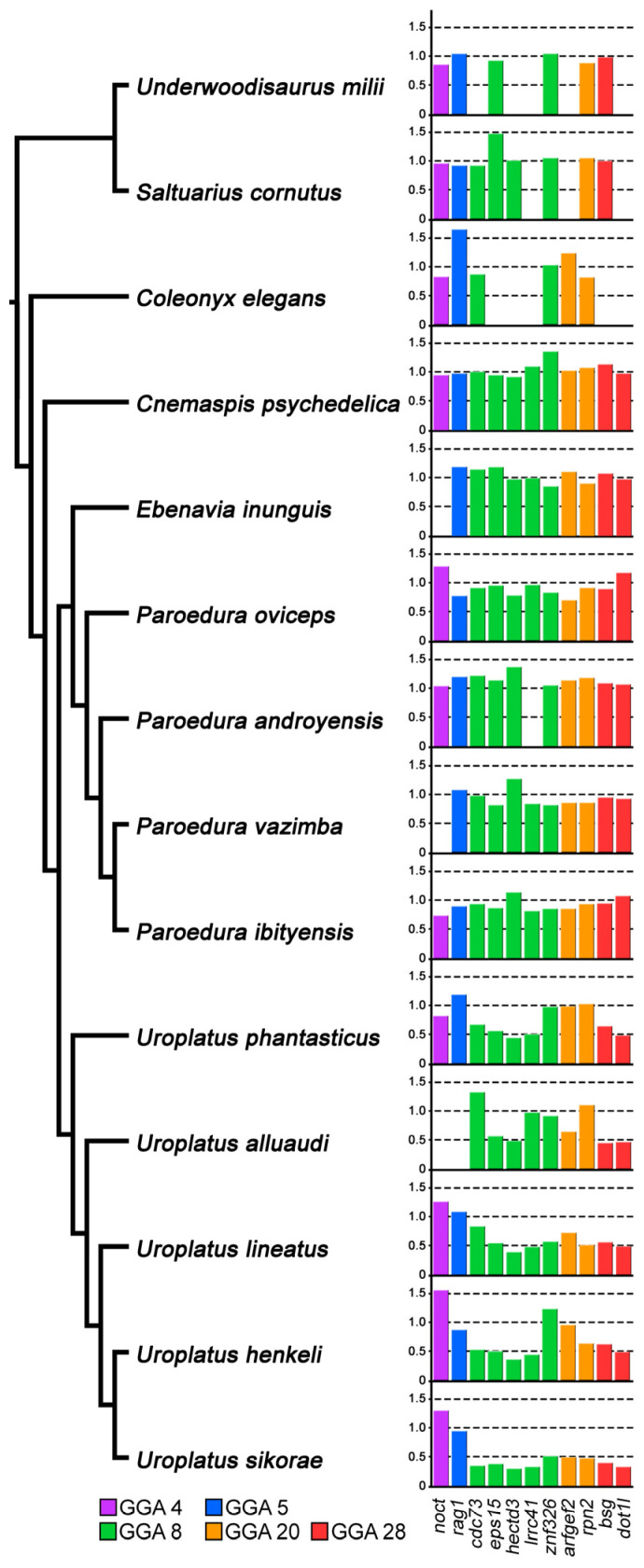
Gene dose ratios between sexes for 11 genes (2 autosomal and 9 linked to Z chromosome in the genus *Uroplatus*) across 14 species of geckos. We expect rations of ∼1.00 for autosomal and pseudoautosomal genes, and ∼0.5 for Z-specific genes. Missing bars indicate that the specific gene was not successfully amplified by qPCR in the given species. Phylogenetic branching patterns are according to [25,53]. All data are available in Table S3.

We designed primers from nine genes with orthologs linked to GGA 8 (*cdc73, eps15, hectd3, lrrc41, znf326*), GGA 20 (*rpn2, arfgef2*) and GGA 28 (*bsg, dot1l*). The qPCR analyses confirmed the results of the gene coverage analysis (Tables S1 and S2): the genes *cdc73*, *eps15*, *hectd3*, *lrrc41*, *rpn2*, *bsg* and *dot1l* are Z-specific, while the genes *znf326* and *arfgef2* are autosomal or pseudoautosomal in *U. henkeli* (Figure 4). The genes *eps15*, *hectd3*, *bsg* and *dot1l* are Z-specific in all five examined species from the genus *Uroplatus*, while the genes *cdc73*, *lrrc41*, *znf326*, *arfgef2* and *rpn2* might occasionally be autosomal or pseudoautosomal in *U. alluaudi, U. lineatus, U. phantasticus* and *U. sikorae* (Figure 4).

## 4. Discussion

Our comparative gene coverage analysis between sexes in *U. henkeli* revealed an extensive Z-specific region with homologs linked to chicken chromosomes 8 (GGA8), 20 (GGA20), 26 (GGA26) and 28 (GGA28) (Figure 3). Our analysis shows that the sex chromosomes in this species are largely non-recombining and that the highly degenerated W chromosome lacks many genes present on the Z chromosome. Among amniotes, some genomic regions tend to turn into sex chromosomes more frequently than others, as recently reviewed in [56]. Among the four genomic regions that appear Z-specific in *U. henkeli* (Figure 3), GGA28 may have the most notable role in sex determination. Its homologs became co-opted as parts of sex chromosomes among amniotes independently at least four times, namely in monotremes [5], varanids, beaded lizards and some anguids [57], in the eublepharid gecko *Coleonyx brevis* [30] and in the *Uroplatus* geckos (this study). This region contains *amh*, the gene that was co-opted as a sex-determining gene in at least five lineages of teleost fishes, recently reviewed by Pan et al. [58]. Among other functions, this gene plays a prominent role in the mitotic activity of germ cells [59], which can explain its tendency to become a sex-determining locus because the number of germ cells seems to contribute to sex-specific gonad development at least in some vertebrate lineages [60]. Currently, the homolog of *amh* is the best candidate for sex-determining locus in monotremes [5], and it will be interesting to explore whether it has the same function in the reptile lineages with sex chromosomes homologous to GGA28.

The Z-specificity of several genes (*eps15*, *hectd3*, *bsg* and *dot1l*) is shared among five species of the genus *Uroplatus*, strongly suggesting that their differentiated sex chromosomes are homologous (Figure 4). The qPCR test for the homology of sex chromosomes revealed that all nine tested genes from the genomic regions 8, 20 and 28 are Z-specific only in *U. sikorae*. On the contrary, some of these genes are pseudoautosomal or autosomal in the other four examined species of the genus *Uroplatus*, which indicates that either (1) these genes were translocated from sex chromosomes to autosomes or to the pseudoautosomal region in these species due to chromosomal rearrangements, or (2) these species have followed different evolutionary strata of sex chromosome differentiation.

The ZZ/ZW sex chromosomes were recently identified by cytogenetic methods in *U. alluaudi* by Mezzasalma et al. [44], who noticed a prominent, heterochromatic and metacentric W chromosome. In this study, we confirm the presence of ZZ/ZW sex determination in *U. alluaudi*, and we provide further cytogenetic evidence for the presence of heterochromatic and acrocentric W chromosomes in *U*. *sikorae* and *U. henkeli* (Figure 1 and Figure 2). Mezzasalma et al. [44] concluded that the Z chromosome is a small acrocentric chromosome corresponding to chromosome pair 10. Nevertheless, we suggest that the Z chromosome is a rather large acrocentric chromosome, corresponding to chromosome pair 3 (or putatively to pair 4) in both *U. alluaudi* and *U. henkeli*. In these two species, the W chromosomes are very prominent in Giemsa-stained metaphases (big metacentric and largest acrocentric, respectively), and therefore, we could assign the pair of sex chromosomes, in contrast to *U*. *sikorae*, where the W chromosome is acrocentric and of similar size to the chromosomes from the pairs 2, 3 and 4. We believe that the difference between our study and Mezzasalma et al. [44] on the assignment of the sex chromosome pair can be explained due to the difficulty of pairing chromosomes in a karyotype with almost only acrocentric chromosomes, gradually decreasing in size. This difficulty was also acknowledged by Mezzasalma et al. [44]. Additional support for our assignment of the Z chromosome as chromosome pair 3 comes from the gene coverage analysis. In fact, the Z-specific region in *U. henkeli* consists of four genomic regions homologous to GGA8, GGA20, GGA26 and GGA28 (Figure 3), which is more consistent with a larger rather than a small-medium chromosome.

Based on chromosome morphology, Mezzasalma et al. [44] suggested that the ZZ/ZW sex chromosomes of *U. alluaudi* are not homologous with the sex chromosomes of other related geckos. The equal gene dose in both sexes of the tested *U. henkeli* Z-specific genes in geckos from other genera supports that the differentiated ZZ/ZW sex chromosomes are probably an apomorphy of the leaf-tail geckos (Figure 4). This conclusion is also in agreement with the differences in the gene content, as the differentiated ZZ/ZW sex chromosomes of some members of the Madagascar genus *Paroedura* share genes with the part of the chicken chromosomes 4 (GGA4) and 15 (GGA15) [40], which are not part of the Z-specific region in the leaf-tailed geckos (Figure 3). The distribution of sexual differences in gene dose across the geckos allows us to trace the origin of the differentiated sex chromosomes of the leaf-tail geckos at least to the last common ancestor of *U. henkeli* and other four studied members of this genus, which lived approximately 40 million years ago [53]. Together with carphodactylid and pygopodid geckos [21,32], the genus *Uroplatus* can be considered as another gekkotan lineage with rather old and stable sex chromosomes.

Within squamates, the geckos have derived karyotypes, where the ancestral squamate microchromosomes mostly fused to form larger chromosomes and many chromosomes are (at least in the putative ancestral gekkotan karyotype) acrocentric [61,62,63]. This situation resembles the independently evolved karyotypes of lacertids, which mostly lack metacentric chromosomes and microchromosomes [63,64,65,66]. In this respect, the karyotypes of the genus *Uroplatus* are typical for geckos; all nine up-to-date cytogenetically examined species possess exclusively acrocentric chromosomes, with a single exception [44] in this study. The only metacentric chromosome found in the genus is the W chromosome of *U. alluaudi*, which is very likely a derived state.

Generally, acrocentric chromosomes can turn into metacentric by (1) interchromosomal rearrangements, mostly Robertsonian fusions, which seem to be quite rare in geckos, in contrast to mammals [61,63]; (2) intrachromosomal rearrangements, i.e., pericentric inversions, which are common in geckos [63]; or (3) accumulations of repeats leading to an expansion of a minute chromosome arm of the ancestral acrocentric chromosome, such as in the voles of the genus *Microtus* [67]. Notably, previous studies have shown that the differentiated Y and W chromosomes are highly dynamic parts of the genome and can differ significantly in size, heterochromatin distribution, gene content and repetitive element content, even between closely related species that share homologous sex chromosomes [5,6,9,68,69,70,71,72,73,74,75]. In *U. alluaudi,* the W is extraordinarily large, it is the largest chromosome in the karyotype, but the same is true also for the acrocentric W chromosome in *U. henkeli* (Figure 1 and Figure 2). We can speculate that the metacentric shape of the W chromosome of *U. alluaudi* formed due to a further accumulation of repeats and/or pericentric inversion in the ancestral, highly heterochromatic W chromosome. This hypothesis should be tested in future by an analysis of synteny across W chromosomes of the leaf-tail geckos. The comparative gene coverage analysis (Figure 3) as well as the highly heteromorphic nature of the W chromosomes (Figure 1 and Figure 2) in the leaf-tail geckos, suggest that their W chromosomes have an extensive non-recombining region. The exceptional shape of the W chromosome in *U. alluaudi* might be connected to the fact that in contrast to the Z chromosomes and the autosomes, which stayed all acrocentric for a long evolutionary time, it mostly does not recombine and thus does not have to pair with its counterpart.

It was expected that the enormous repeat and heterochromatin accumulation is typical for earlier stages of differentiation of sex chromosomes after cessation of recombination, with progressive loss of the heterochromatic and repeat content at later stages leading to diminution of Y and W chromosomes [76,77,78]. In any case, the large size of the W chromosomes in all examined leaf-tail geckos, their large non-recombining region and their rather ancient origin suggest that a size expansion of unpaired sex chromosomes cannot be generally taken as an indication of its recent diversification.

In summary, we documented rather ancient, highly differentiated ZZ/ZW sex chromosomes in the geckos of the genus *Uroplatus*. Their sex chromosomes seem not to be homologous to sex chromosomes of other lineages, but these geckos co-opted the same genomic region for the role of sex chromosomes as at least three other amniote lineages. This independent co-option can be, in the future, used for the exploration of convergent and divergent evolution on sex chromosomes under the control of background of gene content as previously applied to an analogous system, i.e., the green anole *Anolis carolinensis* and the Florida softshell turtle *Apalone ferox* [79].

## Figures and Tables

**Table 1:** List of specimens that were used in the current study.

Family	Species	♂	♀
Carphodactylidae	*Underwoodisaurus milii*	1	1
Carphodactylidae	*Saltuarius cornutus*	1	1
Eublepharidae	*Coleonyx elegans*	1	1
Gekkonidae	*Cnemaspis psychedelica*	1	1
Gekkonidae	*Ebenavia inunguis*	1	1
Gekkonidae	*Paroedura oviceps*	1	1
Gekkonidae	*Paroedura androyensis*	1	1
Gekkonidae	*Paroedura vazimba*	1	1
Gekkonidae	*Paroedura ibityensis*	1	1
Gekkonidae	*Uroplatus phantasticus*	1	1
Gekkonidae	*Uroplatus alluaudi*	1	1
Gekkonidae	*Uroplatus lineatus*	1	1
Gekkonidae	*Uroplatus henkeli*	2	3
Gekkonidae	*Uroplatus sikorae*	1	1

## Data Availability

The raw Illumina reads were deposited in the NCBI database under the BioProject ID PRJNA917835. All other data are provided directly in tables, figures and Supplementary Materials of the manuscript.

## Supplementary Materials

The following supporting information can be downloaded at: https://www.mdpi.com/article/10.3390/cells12020260/s1, Table S1: Parameters for map-to-reference in Geneious Prime.; Table S2: Gene coverage analysis in *U. henkeli.;* Table S3: Primers for 11 tested genes and their gene dose ratios between sexes across 14 species of geckos.
